# The Effects of Oral Isotretinoin in Women with Acne and Polycystic Ovary Syndrome

**DOI:** 10.1155/2019/2513067

**Published:** 2019-04-07

**Authors:** G. Acmaz, L. Cınar, B. Acmaz, H. Aksoy, Yusuf Taner Kafadar, Y. Madendag, F. Ozdemir, E. Sahin, I. Muderris

**Affiliations:** ^1^Erciyes University Hospital, Department of Obstetrics and Gynecology, Kayseri, Turkey; ^2^Erciyes University School of Medicine, Department of Dermatology, Kayseri, Turkey; ^3^Kayseri Education and Research Hospital of Medicine, Department of Internal Medicine, Kayseri, Turkey; ^4^Kayseri Education and Research Hospital of Medicine, Department of Obstetrics and Gynecology, Turkey; ^5^Anamur Government Hospital, Department of Obstetrics and Gynecology, Mersin, Turkey; ^6^Sivas Sarkısla Government Hospital, Department of Obstetrics and Gynecology, Sivas, Turkey

## Abstract

**Introduction:**

Many patients who were diagnosed as polycystic ovary syndrome- (PCOS-) related acne were not capable of sustaining or beginning oral contraceptive pills (OCPs) due to pill scaring, contraindications of OCP use, migraine, or smoking. In this situation, oral isotretinoin treatment may become an important option for PCOS-related acne. The aim of the study was to determine the effects of isotretinoin treatment on PCOS patients who were complicated with severe cystic acne.

**Materials and Methods:**

This study consisted of 40 female patients diagnosed as PCOS complicated with severe cystic acne. These patients were not eligible candidates for OCP use due to migraine, thrombophilia, heavy smoking, or pill scare. To establish baseline values of hormone levels, on days 2–5 of the menstrual cycle, venous blood samples were obtained. Moreover Modified Ferriman-Gallwey (mFG) score, acne score (AS), follicle count, and bilateral ovarian volumes were evaluated both before and after isotretinoin treatment.

**Results:**

Isotretinoin treatment significantly decreased Ferriman-Gallwey score, free testosterone, insulin level, hemoglobin level, acne score, and ovarian volume. Increased triglyceride and cholesterol levels were detected after treatment.

**Conclusion:**

Isotretinoin treatment may have beneficial effects on free testosterone, insulin, acne score, and Ferriman-Gallwey score. Solely isotretinoin administration may supply adequate healing in PCOS patients' symptoms complicated with severe cystic acne who is not eligible candidates for OCP use. This trial is registered with Clinicaltrials.gov NCT02855138.

## 1. Introduction

Polycystic ovary syndrome (PCOS) is the most common endocrine disorder in the female population, with an incidence of 6 to 8 percent. Acne vulgaris (AV) is a well-known feature of PCOS and can complicate approximately 62 percent of PCOS patients during adolescence [1].

Although oral contraceptive pills (OCPs) are widely used, patients may refuse to use OCPs due to adverse effects and/or medical conditions or health care providers may be reluctant to prescribe these drugs because of thrombophilia, migraine, or heavy smoking [2]. Moreover a well-designed study demonstrates that OCP use tends to decrease over time among health care providers. Seaman HE et al. reported that incidence of OCP use decreased 15 to 35 percent in patients with severe acne [3].

The mechanism of isotretinoin action is that it reduces sebum secretion, inhibits bacterial proliferation, inhibits cell proliferation, induces differentiation and apoptosis in different cell types, controls the formation of microcomedones, reduces the formation of lesions and existing comedones, and normalizes desquamation of the epithelium. It may also show anti-inflammatory properties [4–7].

There is growing evidence that isotretinoin's major mode of action in acne is sebocyte apoptosis that is resulting in sebum suppression. Its teratogenicity is also regarded as an apoptotic effect of this retinoid on neural crest cells. It is expressed that isotretinoin enhances the expression of p53 and FoxO1 and FoxO3, all apoptosis-promoting transcription factors [8–11].

Novel studies show that FoxO1 and FoxO3 are p53 target genes for isotretinoin [9]. There is a subgroup of patients who are diagnosed as PCOS complicated with severe cystic acne but not suitable candidates for OCP treatment. Moreover a proportion of PCOS patients refuse OCP treatment due to pill scare migraine, thrombophilia, or smoking but receive isotretinoin because of cosmetic concerns. This study aimed to clarify the outcome of solely isotretinoin treatment on PCOS symptoms.

## 2. Materials and Methods

This study was approved by the Institutional Review Board and the Ethics Committee of the Erciyes University School of Medicine, and all participants signed an informed consent to participate. This prospective study was performed at the Kayseri Education and Research Hospital of Medicine, Department of Obstetrics, and Gynecology and Erciyes University School of Medicine, Department of Dermatology. The clinical trial registration number of this study is NCT02855138.

Our study group consisted of 40 patients with PCOS and acne (aged 18-40 years, BMI, 18-44kg/m2) who attended our obstetrics and gynecology clinic for treatment of AV. All patients in study group were not eligible to use OCPs due to contraindications of medication or pill scaring. All patients underwent detailed physical examination for Modified Ferriman-Gallwey (mFG) score and acne score (AS). Progestin (medroxyprogesterone acetate, 10 mg/day for 10 days) was used to induce menstrual bleeding in PCOS cases with oligomenorrhea/amenorrhea.

To establish baseline values, on days 2–5 of the menstrual cycle, venous blood samples for hormonal assays and other biochemical parameters were obtained in the morning after 6 hours of fasting. Serum free triiodothyronine (fT4), free thyroxin (fT3), thyroid-stimulating hormone (TSH), follicle-stimulating hormone (FSH), luteinizing hormone (LH), estradiol (E2), insulin, insulin-like growth factor-1 (IGF-1), sex hormone binding protein (SHBP), dehydroepiandrosterone (DHEA), free testosterone (fT), and total testosterone (tT) levels were analyzed using an automated chemiluminescence system. Hemoglobin, platelet, and white blood cell concentrations were measured using an automatic hematology analyzer. Serum AST, ALT, cholesterol, triglyceride, and high density lipoprotein levels were measured using an auto analyzer. Sensitivity, specificity, and inter- and intra-assay coefficients of variation were within the limits provided by the manufacturers. After examination as previously described in the literature, the patients were treated with 0.6–0.8 mg/kg oral isotretinoin, up to a total dose of 120–150 mg/kg [12]. Treatment was started at 20 mg/day and gradually increased to the maximum of 40 mg/day. The patients were monitored monthly according to our clinics fallow-up criteria during isotretinoin treatment as previously described [13]. Then all patients were examined for the same parameters after six months.

Exclusion criteria were as follows: (1) presence of any dermatologic disorder besides acne; (2) presence of any systemic disease; (3) pregnancy or lactation; (4) infectious diseases; (6) use of antidepressants, steroidal hormone drugs, mood stabilizers, caffeine, alcohol, or tobacco; and (7) history of ovarian surgery or abdominal surgery for endometriosis.

PCOS was diagnosed according to Rotterdam criteria [14] that includes (1) oligomenorrhea (interval between periods was >35 days) or amenorrhea (absence of vaginal bleeding for 6 months); (2) hyperandrogenism (hirsutism score >7 according to mFG score, acne, and serum testosterone ≥2.7 nmol/l); and (3) polycystic ovaries and exclusion of other PCOS-like syndromes, such as adrenal dysfunction, Cushing syndrome, congenital adrenal hyperplasia, androgen-secreting tumors, enzyme deficiency (21-hydroxylase in particular), hyperprolactinemia, and thyroid dysfunction. Ultrasonographic diagnosis of polycystic ovaries was based on the presence of multiple cysts (≥12 small follicles in each ovary [2-9 mm in diameter] arranged peripherally and scattered throughout the dense core of stroma [the necklace appearance of follicular cysts [and/or increased ovarian volume >10 mL] on pelvic or vaginal ultrasound examination). A medical history was obtained from each patient at the first visit. In order to evaluate the ovarian morphology of patients, pelvic ultrasound was performed by the author (G.A.).

Participants underwent ultrasonographic pelvic assessments on the same day using a Toshiba Xario (Toshiba Medical Systems Corporation, Japan) equipped with a curved array transducer and a 3.5- to 5-MHz convex probe. Ovarian volume was calculated according to the previously described [15]. All patients who met the eligibility criteria were provided isotretinoin treatment and were sequentially recruited by our dermatologist (L.C.).

## 3. Power Analysis

Sample size analysis was made for the two dependent groups (matched pairs). The groups were evaluated for pretreatment ovarian volume left and posttreatment ovarian volume left. We assumed power = 0.80 and alpha = 0.05. Sample size determination was made for double comparison (mean of pre-treatment ovarian volume left versus mean of post-treatment ovarian volume). Sample size reference values (means, standard deviations, and reference sample sizes) were obtained from patient data observed by the authors. Taking into account all these results, our need for a total sample size is 40 for comparison [16]. The analysis was made using G*∗*Power 3.1.7.

## 4. Statistical Analysis

To test the normality assumption of the data, the Shapiro Wilk test was used. Values are expressed as mean ± standard deviation or median (25th–75th percentile). Parametric comparisons were made with t-test for two dependent groups; nonparametric comparisons were made with Wilcoxon Signed Ranks test. The PASW Statistics 18 program was used for all comparisons. Probability value p<0.05 was considered as statistically significant.

## 5. Results

All the women in our study were of reproductive age. The effect of isotretinoin on ovaries, mFG, and acne score is illustrated in Table 1.

We found improved acne and mFG scores and bilaterally decreased ovarian volumes after isotretinoin treatment.

Baseline serum hormone levels and biochemical parameters were evaluated and are shown in Table 2.

We detected significantly decreased fT level; moreover cholesterol and triglyceride levels were increased after isotretinoin treatment but cholesterol and triglyceride levels were within normal limits and did not reach high levels that bloc treatment.

## 6. Discussion

OCPs are well-known treatment options, especially in PCOS-related acne and are largely prescribed worldwide. Seaman et al. reported that many patients are not capable of sustaining or beginning OCPs due to pill scaring, contraindications of OCP use, migraine, or smoking [3]. In this situation, oral isotretinoin treatment may become an important option for PCOS-related acne. On the other hand if oral isotretinoin treatment is used for PCOS-related acne, then how will other findings of PCOS be affected by this treatment modality? As to our knowledge, present study is the first study in the literature which evaluates the outcome of solely isotretinoin treatment on PCOS.

Isotretinoin, which has been used in the treatment of AV, is a synthetic retinoid derivative. It reduces sebum secretion, inhibits bacterial proliferation, inhibits cell proliferation, induces differentiation and apoptosis in different cell types, controls the formation of microcomedones, reduces the formation of lesions and existing comedones, and normalizes desquamation of the epithelium. It may also show anti-inflammatory properties [4–7].

We found amelioration of mFG and acne scores after isotretinoin treatment. This significant finding may be related to markedly decreased levels of fT, but not, however, significantly decreased insulin and IGF levels. It has been shown that insulin and IGF could activate 17-*α*-hydroxylase, which produces 17-hydroxyprogesterone from progesterone declined, so testosterone levels might decrease in the theca cells [17]. Moreover, IGF-1 accelerates sebaceous lipogenesis in glands [18].

Melick examined the effect of isotretinoin treatment on hair follicles; expressed shaft elongation reduced significantly after just the second day of treatment, and 80% of these follicles entered catagen stage on the sixth day of treatment. This ratio was 30% in the control group [19].

On the other hand, androgens show peripheral effects via reversing fT or dihydrotestosterone [20]. We detected significantly decreased mFG and acne scores after isotretinoin treatment which may be related to peripheral effects of isotretinoin. This decrease was emphasized by Karlsson et al., who investigated the nonclassical pathway of androgen formation and concluded that 11-cis retinol dehydrogenase can reduce the androgenic response in peripheral tissues through increasing prostate-specific antigen (PSA) gene production five- to six-fold [21].

All hormonal assessments were done at the follicular phase of the menstrual cycle. We found significant reduction for fT, but fT4, fT3, TSH, FSH, LH, E2, insulin, IGF-1, SHBP, DHEA, and tT levels did not significantly differ after 6-month isotretinoin treatment. Gokalp H et al. investigated the effect of isotretinoin treatment on fT, fT4, fT3, TSH, FSH, LH, and E2 levels with 60 female volunteers. Isotretinoin treatment significantly reduced fT levels. However fT4, fT3, TSH, FSH, LH, and E2 levels were not significantly changed [22].

In a similar study, Karadag et al. investigated the effects of isotretinoin treatment on fT4, fT3, TSH, FSH, LH, and E2 levels and found that tT levels decreased after treatment. However, FSH, LH, and E2 levels were not significantly changed after treatment [23]. Dissimilar results may be related to different study protocols. Karadag et al. studied both male and female volunteers, whereas we examined only PCOS-related acne in female patients.

Isotretinoin reduces serum IGF-1 levels, an effect that may be related to FoxO1-mediated suppression of hepatic growth hormone receptor. IGF-1 controls gonadal and adrenal androgen synthesis and induces 5alpha-reductase, thus stimulating the quantity and quality of androgen receptor (AR) ligands [24]. IGF-1 via activation of the kinase AKT controls the nucleo-cytoplasmic distribution of the transcription factors FoxO1 and FoxO3. Notably, FoxO1 is a negative nuclear regulator of AR and reduces AR transactivation [25]. p53 is a negative transcriptional regulator of AR gene expression [26]. It is thus not surprising that isotretinoin reduces AR gene expression and transactivation as observed in isotretinoin-treated acne patients [27].

In PCO syndrome, metformin is a very effective agent that is also very valuable for the treatment of resistant acne and acne tarda [28]. It has been demonstrated that metformin treatment increased p53 expression in PCO patients [29]. There is good reason to believe that isotretinoin, as a potent inducer of p53, operates in a similar manner [9].

Huang XF and LuuThe V illustrated that retinol could inhibit oxidative 3*α*-hydroxysteroid dehydrogenase (3*α*-HSD) activity of Retinol dehydrogenase-4 (RoDH-4). Because 3*α*-HSD is an accepted key enzyme in androgen biosynthesis, both authors offer a supportive background for our findings and explain reduced testosterone levels [30].

We reviewed ovarian volume and AFC as another parameter and illustrated a significant decrease in ovarian volume and AFC after isotretinoin treatment. Previous studies showed both antiproliferative and antiangiogenic effects of isotretinoin [31]. Moreover isotretinoin's reported apoptotic effects on rat granulosa cells [32].

There is recent evidence that p53 plays a key role in the regulation of ovarian granulosa cells [32]. It was not known whether the loss in volume was secondary to the decrease of AF via affecting granulosa cells or the antiproliferative effect of isotretinoin on stromal cells of the ovary. If it is related to reduction of stromal cells, it may explain the decrease in serum free testosterone levels.

## 7. Conclusion

Solely isotretinoin treatment may be beneficial in patients with PCOS and acne who are not capable of using OCPs. Large-scale and well-balanced studies are required to understand the effect of isotretinoin in these patients.

## Figures and Tables

**Table 1:** Evaluation of ovarian reserve, hirsutism, and acne scoring.

	Pre-treatment n=40	Post-treatment n=40	*P*-value
Modified Ferriman-Gallwey Score	11,75 ± 4,07	11,08 ± 3,74	0,011
Acne Scoring	2(2-3)	0(0-0,75)	<0,001
Ovarian volume right	11(8-13)	9(7-11,75)	0,026
Ovarian volume left	10,5(8-13,75)	8(7-10,75)	0,018
Follicle count right	10(9-11)	7(6,25-8)	<0,001
Follicle count left	9,88 ± 2,28	6,3 ± 1,71	<0,001

To test the normality assumption of the data, Shapiro Wilk was used. Values are expressed as mean ± standard deviation or median (25th percentile – 75 percentile). Parametric comparisons were made with t-test for two dependent groups; non-parametric comparisons were made with Wilcoxon Signed Ranks test. PASW Statistics 18 program was used for all comparisons. p<0.05 probability value was considered as statistically significant.

**Table 2:** Comparisons of pre- and post-treatment hormone and biochemical parameters.

	Pre-treatment n=40	Post-treatment n=40	*P*-value
FSH	7,005(6,0825-8,24)	7(6,195-8,265)	0,906
LH	5,07(3,645-7,89)	6,09(4,38-8,64)	0,243
E2	43,5(34,25-64,25)	42(33,5-53,75)	0,416
Free-Testosterone	1,79(1,35-2,35)	1,51(1,21-2,01)	0,024
Total-Testosterone	0,435(0,332-0,575)	0,455(0,302-0,57)	0,965
SHBG	39(32,25-44)	37,5(32,5-50,75)	0,482
DHEASO4	197,65(160,67-274,55)	234,6(212,07-271,17)	0,104
IGF1	318,9±68,51	313,25±85,52	0,737
Insulin	10,19(6,29-12,17)	7,70(6,52-10,01)	0,062
Hemoglobin	12,77±0,99	12,50±0,82	0,009
PLT	306,6±60,23	310,9±62,47	0,499
AST	23(19-28)	24(20-28)	0,275
ALT	22(19-26)	25(21-26,75)	0,257
Triglyceride	121,5(103,5-132)	135(119,25-172)	<0,001
Cholesterol	133,23±20,39	153,32±27,32	<0,001
HDL	38,05±6,04	37,48±5,72	0,475

To test the normality assumption of the data, Shapiro Wilk was used. Values are expressed as mean ± standard deviation or median (25th percentile – 75 percentile). Parametric comparisons were made with t-test for two dependent groups; non-parametric comparisons were made with Wilcoxon Signed Ranks test. PASW Statistics 18 program was used for all comparisons. p<0.05 probability value was considered as statistically significant.

## Data Availability

The data used to support the findings of this study are included within the article.
